# Development and initial validation of a defensive pressure index using tracking data in the Chinese super league

**DOI:** 10.3389/fspor.2026.1833549

**Published:** 2026-05-26

**Authors:** Jieying Li, Fang Wu, Ang Li, Raúl Martínez-Santos

**Affiliations:** 1 Faculty of Education and Sport, University of the Basque Country, UPV/EHU, Vitoria-Gasteiz, Spain; 2 Department of Physical Education and Research, China University of Mining and Technology, Beijing, China; 3 Faculty of Science and Technology, Paris–Saclay University, Paris, France

**Keywords:** defensive organization, football analytics, match status, partial temporal analysis, tactical compactness

## Abstract

Tracking data have enhanced the analysis of football performance; however, defensive pressure remains insufficiently operationalised. This study aimed to develop a Defensive Pressure Index (DPI) based on movement tracking data, to examine its association with defensive outcomes, and to explore whether DPI differs across running score. Positional tracking data from 30 Chinese Super League matches during the 2018 season were analysed, yielding 2,032 defensive sequences. Defensive pressure was conceptualised as the degree of spatiotemporal constraint imposed on the attacking team during defensive sequences and was quantified using a composite index derived from six spatial tactical variables. Ordered logistic regression was used to examine the association between DPI and defensive outcomes, while one-way ANOVA was used to assess differences in DPI across running score. Higher DPI values were significantly associated with more favourable defensive outcomes (*p* < 0.001), providing preliminary evidence that the index captures meaningful sequence-level variation in defensive pressure. However, this finding should be interpreted with caution because the proportional odds assumption showed evidence of deviation. By contrast, DPI did not vary significantly across running score, suggesting that defensive pressure at the sequence level was more closely related to local spatial tactical organization than to scoreline alone. Overall, the DPI provides a transparent and replicable framework for quantifying defensive pressure in professional football. Future research should further evaluate the index using broader datasets and modelling approaches that account for the hierarchical structure of match data.

## 1 Introduction

Football action is structured around the collective objectives of two teams, each aiming to score more goals than the opposing squad. In this sense, the outcome of a match represents the ultimate social performance indicator in the sport (1). Within this competitive context, offensive and defensive processes are inherently interdependent, as each team continually seeks to overcome the opponent's efforts while preventing goalscoring opportunities. As an interactive team sport, football performance emerges not only from individual technical actions but also from the dynamic, intelligent coordination of players within spatial and temporal constraints (2–4). From a temporal perspective, football matches can be conceptualized as a continuous interaction of four interrelated phases: organised attack, organised defense, offensive transition following ball recovery, and defensive transition following ball loss (5).

These phases capture the evolving tactical structure of the game and provide a useful framework for analyzing team behaviors. With the increasing availability of tracking data, performance analysis in football has shifted towards more objective and finer grained approaches (6, 7). Tracking data enable the quantification of collective behaviors and spatial interactions between teams, providing new opportunities to analyze tactical organization and decision making processes (8, 9). Although substantial research has focused on attacking behaviors (10), physical characteristics (11–13), and technical actions (14, 15), defensive processes particularly during organised defensive phases and transitions remain comparatively underexplored. Little is known about how defensive pressure emerges and is maintained over time, as much of the existing literature assesses defensive performance using indirect or outcome based indicators.

Traditional defensive metrics, such as tackles, interceptions, fouls, or goals conceded, fail to capture the dynamic and collective nature of defensive behaviors (16, 17). These indicators do not adequately represent defensive pressure, which reflects both the immediate constraints imposed on the ball carrier and the broader spatial organization of the defending team (18, 19). Moreover, previous studies have examined defensive behaviors from multiple perspectives. For instance, some research has shown that teams adapt their defensive strategies by modifying spatial occupation and player synchronization (20), while different tactical formations (e.g., 4-4-2 vs. 5-3-2) can influence defensive organization and pressing behaviors (21). Other studies have focused on specific defensive strategies such as high pressing, counter pressing, and ball recovery dynamics (22–24), and Castellano and Pic (25) highlighted the distinction between high pressure and deep defensive strategies, while Santos et al. (26) demonstrated how pressing zones vary across tactical contexts.

As a result, there may be growing interest in developing more refined approaches to quantify defensive pressure using positional data. Several attempts have also been made to operationalize defensive pressure. Tenga et al. (27) categorized pressure into different qualitative levels, while Fernandez-Navarro et al. (28) quantified pressure using distances between attackers and defenders. More recently, studies using tracking data have emphasized the importance of spatial compactness and collective positioning in shaping defensive effectiveness (29). Despite these contributions, the concept of defensive pressure remains inconsistently defined, and there is still a lack of unified, replicable frameworks that integrate multiple spatial tactical variables into a comprehensive measure (18). In this sense, recent research has further emphasized the importance of spatial context in shaping defensive outcomes. For example, Jamil et al. (30) demonstrated that the effectiveness of organised pressing is strongly influenced by the location of ball recovery, suggesting that the place where possession is regained plays a critical role in determining subsequent defensive success.

Building on this perspective, the present study extends previous work by integrating multiple spatial tactical dimensions into a composite index (DPI) derived from tracking data, thereby providing a more comprehensive representation of defensive pressure beyond single variable approaches.

Moreover, existing research presents a degree of conceptual ambiguity regarding defensive pressure. In some studies, pressure is treated as a tactical strategy (e.g., pressing systems), while in others it is described as an emergent property reflecting intensity or spatial constraint (19). This lack of conceptual clarity limits the comparability of findings and highlights the need for more systematic approaches to defining and measuring defensive pressure.

Importantly, most empirical studies on defensive behaviors have been conducted in European leagues, with relatively limited research focusing on the Chinese Super League (CSL) (31, 32). Although some studies have examined defensive playing styles in the CSL, phase-level analyses based on tracking data, particularly those addressing defensive pressure, remain scarce. Consequently, the spatial characteristics and determinants of defensive pressure in this context remain poorly understood. In addition, while prior research has identified various defensive patterns and strategies, it remains unclear which specific spatial mechanisms most strongly contribute to the emergence of defensive pressure during defensive sequences. Specifically, the relative importance of factors such as regain location, defensive line positioning, and local player density has not been systematically examined within a unified analytical framework.

Therefore, to address these gaps, the present study focuses on the Chinese Super League and adopts a phase level approach based on positional tracking data. By constructing a composite Defensive Pressure Index (DPI) and evaluating its associations with defensive outcomes and match context, this study aims to provide a more transparent and replicable framework for analyzing defensive pressure. Specifically, the aims of this study were 1) to construct a tracking-data-based Defensive Pressure Index (DPI), 2) to evaluate whether the DPI is meaningfully associated with defensive outcomes, and 3) to examine whether the DPI varies according to the running score.

## 2 Methods

### 2.1 Sample and variables

This study adopted an observational, retrospective, and analytical design based on positional tracking data from official Chinese Super League matches. A total of 2,032 defensive sequences from 30 matches in the 2018 Chinese Super League (CSL) season, involving 16 participating teams, could be analysed. All data were collected through synchronized position tracking, event annotation and systematic recording. Each observation unit was defined as a defensive sequence, beginning when the opposing team gained possession of the ball and ending when the defending team regained possession, or the attacking team lost control of the ball.

To investigate the characteristics of defensive pressure, only organised attacking sequences were included in the analysis, that is, phases during which the attacking team maintained purposeful possession through a series of technical actions. Following the definitions proposed by (28, 33), an attacking team was considered to be in possession under either of the following conditions: the attacking team completed at least three consecutive passes during the same attacking phase, and the attacking player performed a dribbling action lasting at least three seconds. These criteria were adopted to exclude unintentional or transient possessions, such as single passes, unforced errors, or incidental ball contacts.

A defensive sequence was deemed to end when the defending team regained possession. The termination of a sequence could occur under any of the following circumstances:

1. The attacking team played the ball out of bounds.
2. Any attacking player was caught in an offside position.
3. The attacking team committed a foul during possession.
4. If an attacking player passed the ball to a teammate positioned beyond the offside line, and that teammate subsequently advanced and concluded the attack with either a shot or an entry into the defending team's penalty area, the defensive sequence was also considered complete.

**Figure 1:**
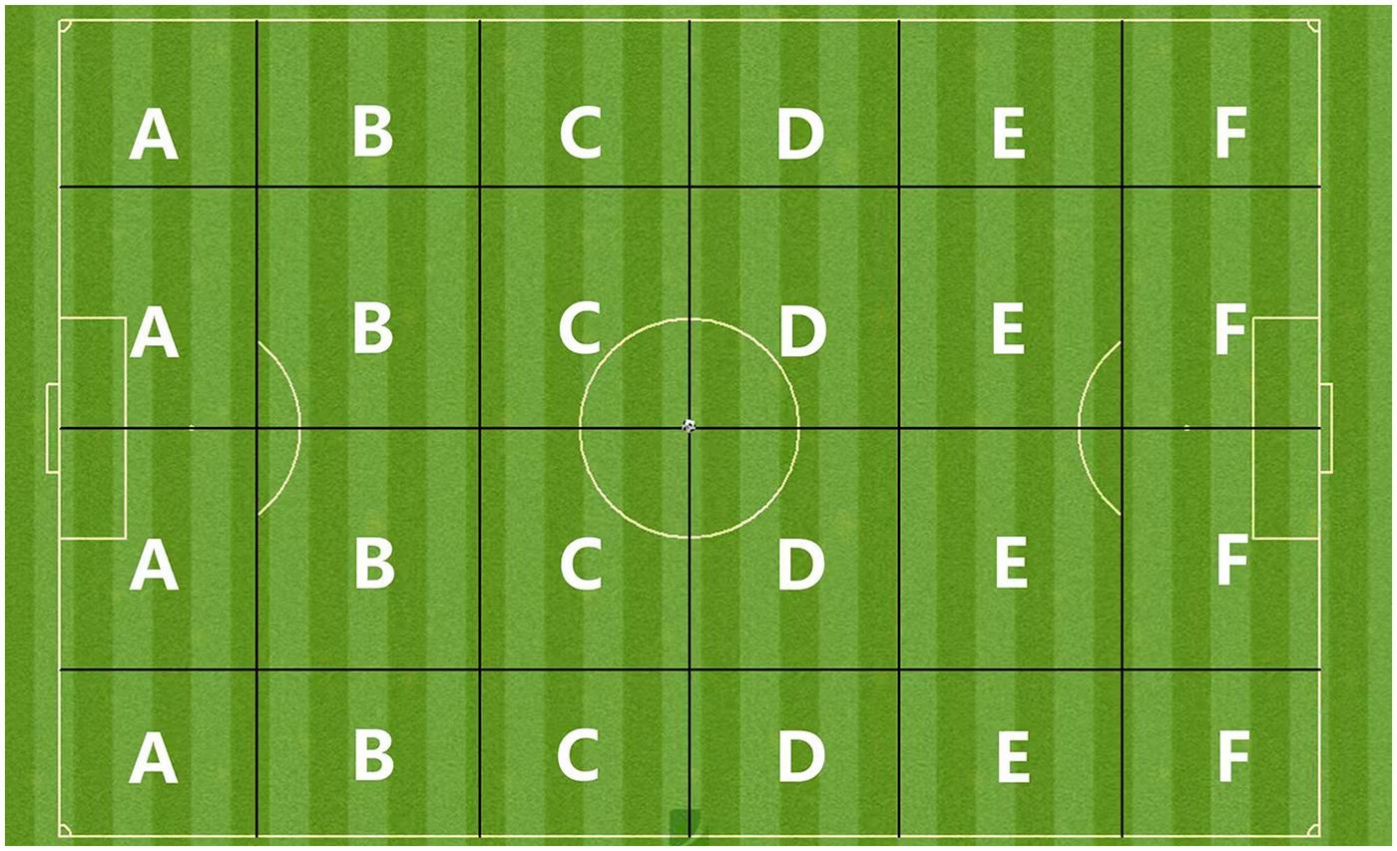
Areas on a football pitch in the amisco Pro® system. DPGZ: The zones labelled from A to F represent the regions where possession is regained, ranging from the area closest to the defending team's goal **(A)** to the area closest to the opponent's goal **(F).**

**Figure 2:**
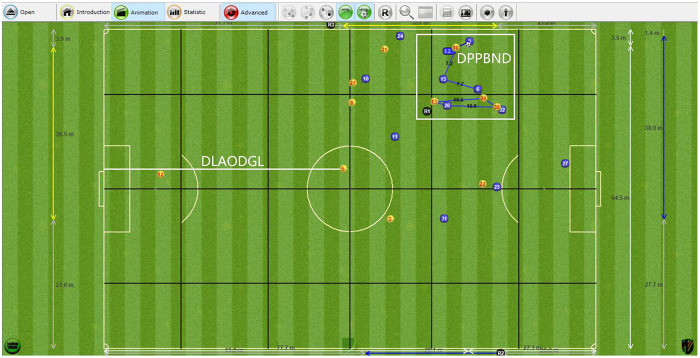
DPPBND and DLAODGL on the football field. DPPBND Indicates the distance from the ball carrier to the nearest defender, marking the pressing intensity; DLAODGL: Reflects the depth of the defensive line and vertical compactness of the defense.

The variables adopted in this study are summarized in Table 1. Based on the definitions proposed in Fernandez-Navarro et al. (28), these variables collectively capture multiple spatial tactical dimensions of defensive pressure. DPGZ: Reflects the position where possession is regained, indicating defensive proactivity and pressing spatial positioning (Figure 1); DLAODGL: Reflects defensive line height, indicating the degree of vertical compression; DPPBND: Reflects the immediate pressing intensity applied to the ball carrier (Figure 2); DNUM: Reflects the number of players involved in localized pressing/Localized support; PL, PN: Reflect offensive progression characteristics, serving as contextual or contest process variables.

**Table 1:** Operational definitions, extraction procedures, and tactical rationale of the measured variables.

Variable	Operational definition	Tactical meaning
DPGZ	Pitch zone in which the defending team regains possession, coded from Zone A (closest to own goal) to Zone F (closest to opponent's goal).	Indicates defensive proactivity and territorial location of ball recovery.
DPPBND	Distance between the attacking ball carrier and the nearest defender.	Indicates the intensity of direct pressure on the player in possession.
DLAODGL	Distance from the least advanced outfield defender to the own goal line.	Indicates defensive line depth and vertical compactness.
PL	Distance of the final attacking pass before possession is lost or the sequence ends.	Reflects the opponent's immediate progression pattern.
DNUM	Number of defenders located within a predefined local area around the ball carrier.	Reflects local defensive density and collective support near the ball.
PN	Number of passes completed by the attacking team before sequence termination.	Reflects the extent of attacking circulation before defensive interruption.
Running Score	Match-status category at the time of the sequence: losing, drawing, and winning.	Represents the contextual score state affecting defensive behaviors.
Outcome	Defensive sequence results coded as F, GP, or CDS.	Represents the final defensive performance outcome of the sequence.

Outcome categories: F, Fail; GP, gain possession; CDS, continue defensive sequence.

### 2.2 Procedure

**Figure 3:**
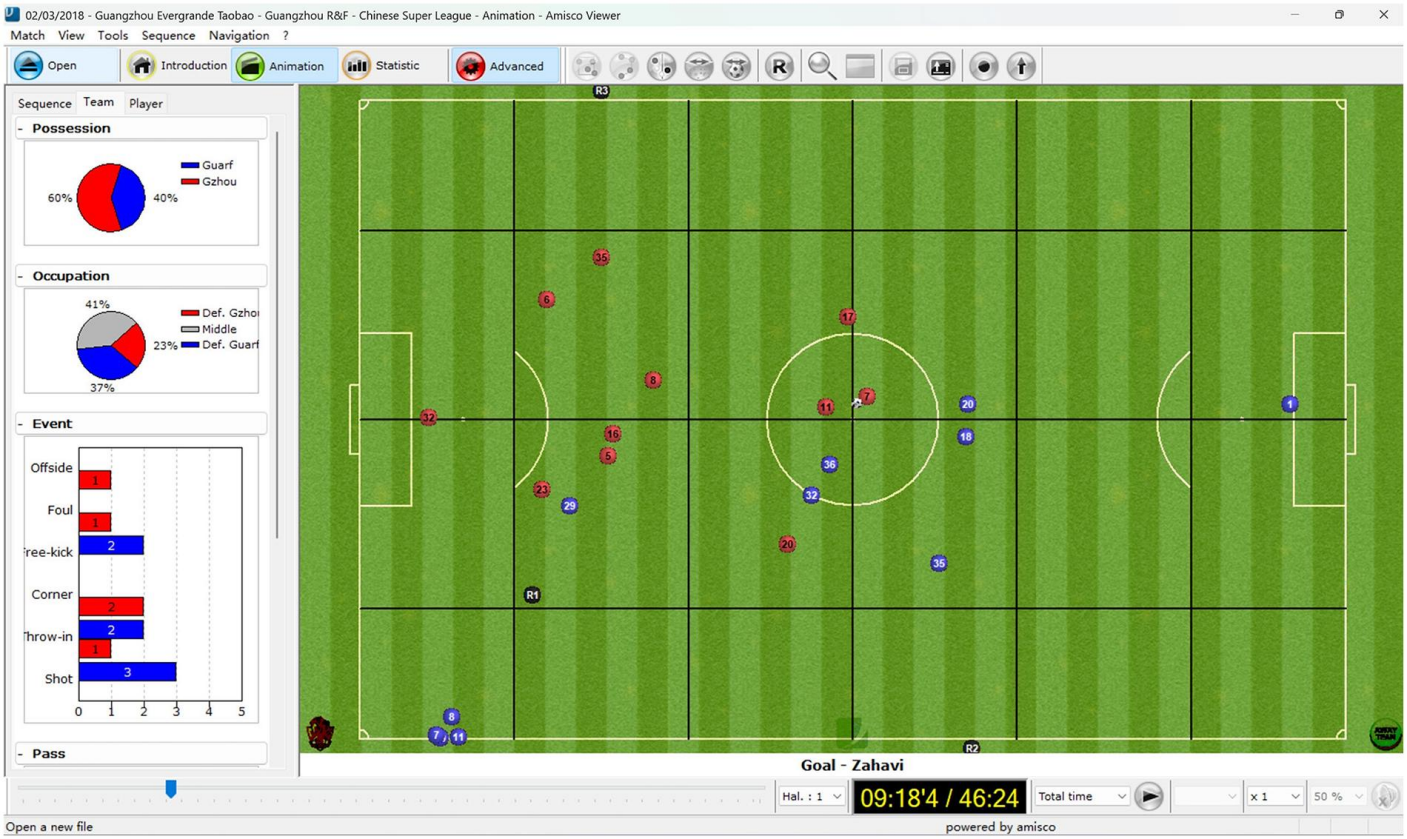
Animation mode of the amisco Pro® system.

The data for this study were generated using the Amisco® tracking system (Amisco®, Nice, France). Written permission from Amisco was obtained before the data collection. This system can precisely track the ball and all players throughout the entire match and generate a two dimensional animation model of player movements, enabling the quantitative analysis of both team and individual performances. All raw data from each game were stored in the computer system for subsequent post-match analysis. The data extraction process was conducted using the Amisco Viewer software (Figure 3). The validity and reliability of this system have been confirmed in previous studies (34, 35).

To ensure consistency between tracking data and defensive sequence event records, this study implemented a systematic data validation procedure. Two trained observers participated in the event annotation process. Prior to formal coding, both observers underwent systematic training to familiarize themselves with the tracking interface, coding procedures, and the operational definitions of all variables.

Following training, reliability testing was conducted to assess both intra-observer and inter-observer consistency. A randomly selected subsample representing 10% of the total dataset (*n* = 203 defensive sequences) was used for repeated coding. For intra-observer reliability, one observer re-coded the selected sequences after a two week interval. For inter-observer reliability, both observers independently coded the same subsample, and their coding results were subsequently compared.

For categorical variables, percentage agreement and Cohen's kappa coefficient were calculated. The inter observer agreement for defensive sequence outcomes was 91% (36), indicating high consistency between different observers. The corresponding Cohen's kappa coefficient was 0.78, indicating “good” agreement according to (37). The intra observer agreement (consistency of repeated assessments by the same observer) was 88%, with a Cohen's kappa of 0.74, demonstrating good reliability within observers.

For continuous variables (DPGZ, DPPBND, DNUM, PL, PN, and DLAODGL), reliability was assessed using intraclass correlation coefficients (ICC), Pearson correlation coefficients (r), and the typical error of measurement (TEM), following the methodology proposed by (38, 39). ICC values were calculated using a two-way mixed effects model for absolute agreement. These metrics collectively demonstrated strong repeatability and measurement precision. All reliability results are presented in Table 2. Overall, the validation procedures ensured the dataset was sufficiently reliable, providing a robust foundation for subsequent quantitative analysis.

**Table 2:** Reliability of continuous tactical variables.

Variable	Mean ± SD	ICC	r	TEM
DPGZ	4.54 ± 1.25	0.92	0.92	0.36
DPPBND	2.48 ± 2.13	0.97	0.98	0.34
DNUM	2.38 ± 1.05	0.83	0.83	0.44
PL	21.35 ± 11.22	0.98	0.98	1.43
PN	5.23 ± 3.82	0.97	0.97	0.60
DLAODGL	62.27 ± 13.21	0.98	0.98	1.91

ICC, intraclass correlation coefficient; r, Pearson correlation coefficient between repeated measurements; TEM, typical error of measurement, representing within observer measurement error based on repeated coding. ICC values were calculated using a two-way mixed effects model for absolute agreement.

### 2.3 Data processing

All variables were standardised using z-score normalisation (mean = 0, SD = 1) to remove differences in scale and ensure comparability across measures (40).

Prior to index construction, each variable was aligned according to its theoretical relationship with defensive pressure. Variables representing greater defensive intensity or spatial constraint (e.g., defensive density and territorial compression) contributed positively to the index, whereas variables associated with reduced defensive pressure (e.g., greater distance to the ball carrier or longer attacking sequences) contributed negatively. Accordingly, higher DPI values consistently indicate greater defensive pressure.

### 2.4 Variable definition and DPI construction

The Defensive Pressure Index (DPI) was defined as a composite indicator to quantify the degree of spatiotemporal constraint imposed by the defending team during a defensive sequence. As summarized in Table 1, the index was constructed from six spatial tactical variables: defensive possession gain zone (DPGZ), distance between the ball carrier and the nearest defender (DPPBND), number of defenders located around the ball carrier (DNUM), pass length of the final attacking pass before sequence termination (PL), number of attacking passes completed before sequence termination (PN), and distance from the least advanced outfield defender to the own goal line (DLAODGL).

These variables capture complementary aspects of defensive pressure, including territorial regain location, immediate pressure on the ball carrier, local defensive density, opponent progression patterns, attacking circulation prior to interruption, and defensive line depth. Accordingly, the DPI was designed to represent defensive pressure as a multidimensional, sequence level construct.

An equal weight formulation was adopted to ensure transparency and interpretability. This approach avoids reliance on sample specific weighting schemes and is consistent with the exploratory PCA results, which did not support a sufficiently strong latent structure for data driven weighting.

The DPI for each defensive sequence was computed as follows, where higher DPI values indicate greater defensive pressure:

$$\mathrm{Defensive}\;\mathrm{Pressure}\;\mathrm{Index}=-Z(DPGZ_{i})-Z(DPPBND_{i})+Z(DNUM_{i})+Z(PL_{i})-Z(PN_{i})+Z(DLAODGL_{i})$$

### 2.5 Statistical analysis

#### 2.5.1 Overview

The statistical analysis was designed to evaluate the Defensive Pressure Index (DPI) as an integrated measure of defensive pressure. Accordingly, the analytical framework tested whether the DPI was associated with defensive outcomes and match context, with subsequent models specified at the level of the composite index rather than its individual component variables.

#### 2.5.2 Exploratory principal component analysis

An exploratory principal component analysis (PCA) was performed to examine the structure of the six spatial tactical variables included in the DPI (41).

The Kaiser-Meyer-Olkin (KMO) statistic was used to assess sample suitability, and Bartlett's sphericity test was conducted to check the appropriateness of the correlation matrix for factor analysis. The KMO statistic suggested some limitations in sample adequacy, while the Bartlett test indicated significant correlations between the variables (42, 43). Factors were extracted based on the Kaiser criterion (eigenvalues > 1), and multiple factors were retained.

#### 2.5.3 Ordinal logistic regression analysis of the defensive pressure index (DPI)

An ordinal logistic regression model (44) was specified to examine whether the Defensive Pressure Index (DPI) was associated with defensive outcomes. This approach was considered appropriate because defensive outcome was treated as an ordered categorical variable with three levels: Fail (F), Gain Possession (GP), and Continue Defensive Sequence (CDS), reflecting increasing levels of defensive effectiveness within a sequence.

The DPI was entered as the sole predictor to evaluate the association between the composite index and defensive outcomes at the sequence level. Parameter estimates were interpreted as odds ratios, indicating the change in the odds of achieving a higher level defensive outcome category for a one-unit increase in DPI. Model fit was summarized using McFadden's pseudo-R², and the proportional odds assumption was assessed using the Brant test prior to interpretation.

#### 2.5.4 Match context analysis

To examine whether defensive pressure varied according to match context, a one-way analysis of variance (ANOVA) was conducted with running score (losing, drawing, winning) as the independent variable and the Defensive Pressure Index (DPI) as the dependent variable. *post hoc* pairwise comparisons were performed using Tukey's HSD test. Effect size was estimated using eta squared *η*^2^ (45).

#### 2.5.5 Software and significance level

All statistical analyses were performed in R version 4.5.2. Excel data were imported using readxl, and data processing and cleaning were conducted using dplyr and janitor. Descriptive statistics and reliability summaries were calculated using psych and DescTools. Ordinal logistic regression was performed using MASS, McFadden's pseudo-*R*^2^ was calculated using pscl, and the proportional odds assumption was assessed using brant. Analysis of variance and Tukey's *post hoc* comparisons were conducted using base R functions. Figures were created using ggplot2 and fact extra. Statistical significance was set at *p* < 0.05.

## 3 Results

### 3.1 Exploratory structure of defensive variables (PCA)

As a supplementary exploratory check, a principal component analysis (PCA) was conducted on the six spatial tactical variables. Sampling adequacy was limited (KMO = 0.51), although Bartlett's test of sphericity was significant, *χ*^2^(15) = 344.37, *p* < 0.001.

**Figure 4:**
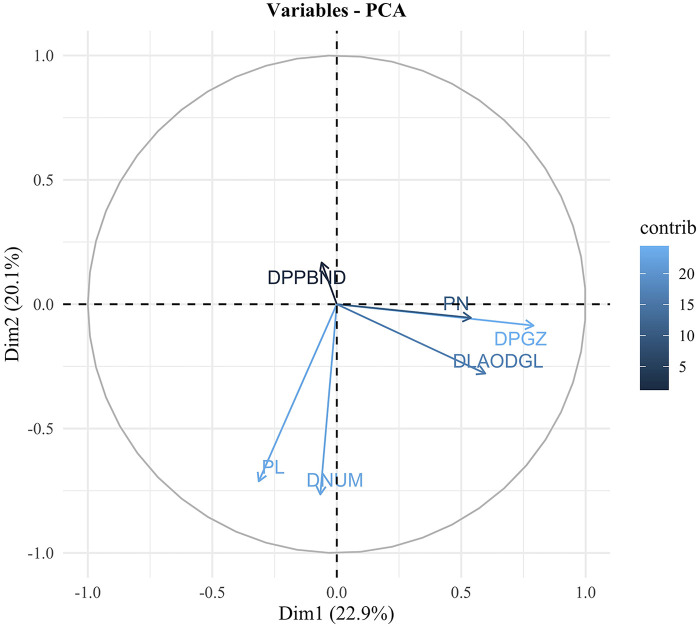
Exploratory PCA of DPI variables; weak factor structure, two components explain 43% of variance.

Two components had eigenvalues greater than 1 and together explained 43.08% of the total variance (see Figure 4). However, the component structure did not reveal a clear low-dimensional pattern. Therefore, the PCA was interpreted for exploratory purposes only and was not used for variable reduction, weighting, or subsequent modelling. Detailed PCA outputs are provided in the Supplementary Materials.

### 3.2 Validation of the defensive pressure Index (DPI)

The ordinal logistic regression analysis (see Table 3) provided preliminary evidence of an association between the Defensive Pressure Index (DPI) and defensive outcomes. Higher DPI values were associated with a greater likelihood of achieving higher-level defensive outcomes. Specifically, a one-unit increase in DPI was associated with an increase in the odds of attaining a higher defensive outcome category (*β* = 0.298, SE = 0.019, z = 15.77, *p* < 0.001), corresponding to a 34.7% increase in odds (OR = 1.347, 95% CI [1.298, 1.398).

**Table 3:** Relationship between DPI and defensive outcomes.

Panel A. Descriptive values						
DPI		F		GP		CDS
−11.7198		0.8725		0.1226		0.0049
−11.5117		0.8654		0.1293		0.0053
−11.3036		0.8581		0.1363		0.0056
−11.0955		0.8503		0.1437		0.0060
−10.8874		0.8423		0.1514		0.0063
−10.6793		0.8338		0.1594		0.0067

Panel B. Ordinal logistic regression results						
Predictor	β	SE	z	*p*	OR	95% CI
DPI	0.2978	0.0189	15.7742	<0.001	1.3469	1.2979, 1.3976

Panel A shows descriptive statistics for DPI, F, GP, and CDS. Panel B indicates that higher DPI is significantly linked to better defensive outcomes (*p* < 0.001, OR = 1.31). McFadden's pseudo-*R*^2^ = 0.0738, meaning the model explains 7% of the variance.

The model demonstrated modest explanatory power (McFadden's pseudo-*R*^2^ = 0.0738), indicating that while DPI captures part of the variation in defensive outcomes, additional contextual and tactical factors are likely to play a role.

The proportional odds assumption was assessed using the Brant test and showed some evidence of deviation (*p* < 0.001), suggesting that the effect of DPI may not be entirely constant across outcome thresholds. Therefore, the results should be interpreted with appropriate caution.

Overall, these findings suggest that the DPI provides a useful sequence level summary of defensive pressure, while further validation using alternative modelling approaches is warranted.

### 3.3 Effect of running score on defensive pressure

To examine whether defensive pressure varied according to match context, a one-way analysis of variance (ANOVA) was conducted with running score (losing, drawing, winning) as the independent variable and the Defensive Pressure Index (DPI) as the dependent variable. Tukey's HSD test was used for *post hoc* comparisons, and effect size was estimated using eta squared (*η*^2^).

The analysis indicated that running score did not have a statistically significant effect on DPI, F(2, 2029) = 0.69, *p* = 0.504, *η*^2^ = 0.001. Mean DPI values showed only minor differences across running score (see Table 4),and Tukey's HSD *post hoc* tests revealed no significant pairwise differences between conditions (*p* > 0.49).

**Table 4:** Running score differences in DPI (updated data).

Running score	*N*	DF	Sum Sq	Mean Sq	F	Pr(>F)
Losing	546	2	11	5.297	0.685	0.504
Drawing	816				0.685	
Winning	670				0.685	
Residuals	2,029		15,692	7.734		
Tukey multiple comparisons of means (95% family-wise confidence level)						
Comparison	diff		lwr	upr		p adj
Drawing-Losing	−0.0582		−0.41885	0.30242		0.9240
Winning-Losing	−0.1802		−0.55627	0.19586		0.4994
Winning-Drawing	−0.12199		−0.46205	0.21807		0.6773

Mean DPI ± SD for each Running Score; Tukey *post hoc* comparisons; no significant differences observed.

**Figure 5:**
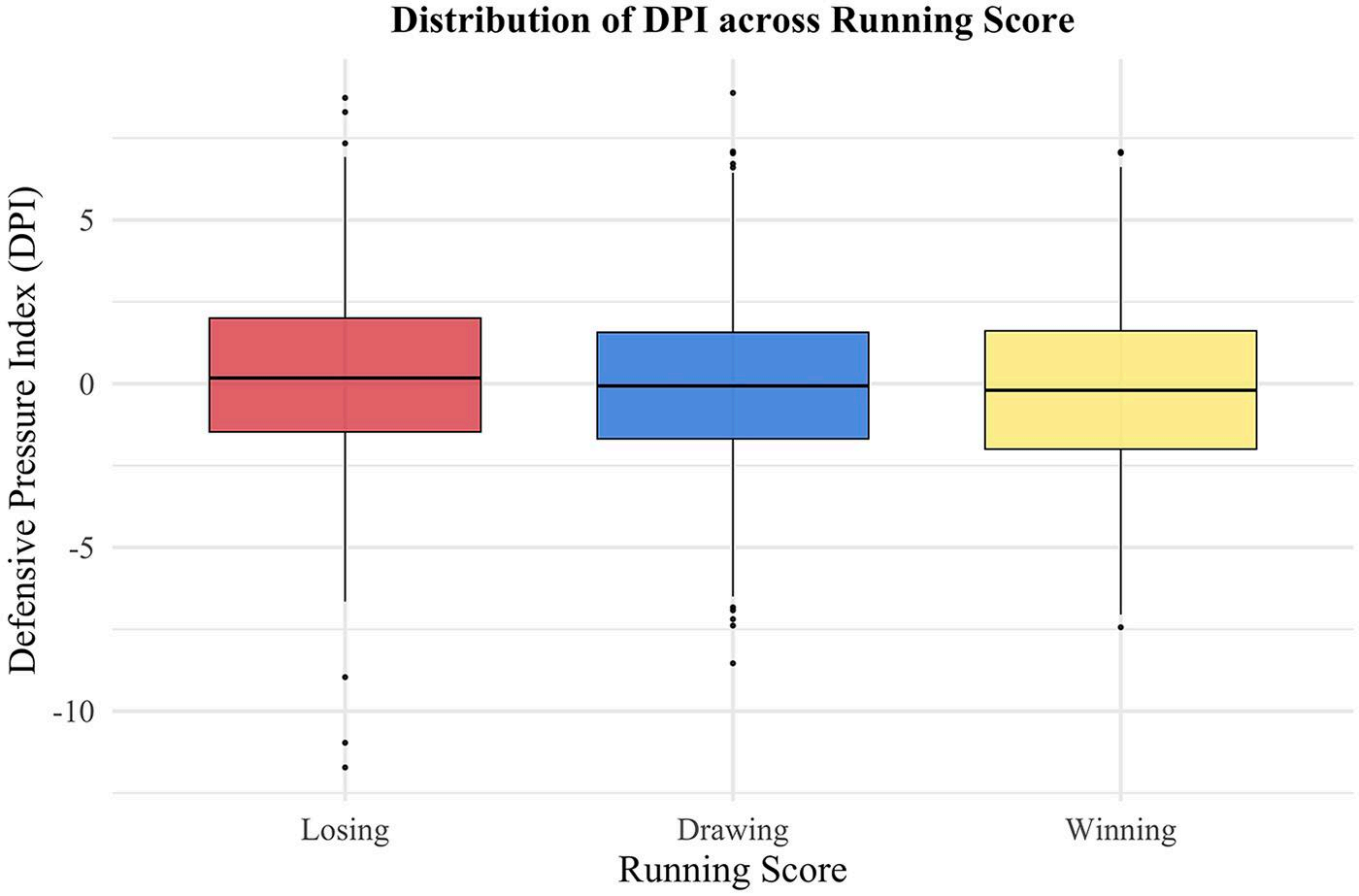
Distribution of the defensive pressure Index (DPI) across running score (losing, drawing, and winning). Higher DPI values indicate greater defensive pressure.

These findings suggest that defensive pressure, as captured by the DPI, does not vary systematically across different running score at the sequence level (see Figure 5).

## 4 Discussion

The present study aimed to quantify defensive pressure using tracking data and to develop a composite measure, the Defensive Pressure Index (DPI), to capture its multidimensional nature in Chinese Super League (CSL) matches. Overall, the findings suggest that defensive pressure, as operationalised by the DPI, is not solely the result of isolated defensive actions but rather emerges from the collective spatial organization of the defending team. In this sense, the study shifts the analytical focus from individual spatial tactical variables to an integrated index, allowing defensive pressure to be represented as a more coherent and operational construct, which is important given the complexity of team sports.

Previous research has highlighted the importance of spatial tactical factors in shaping defensive pressure (18, 19, 46, 47). The present study extends this line of work by combining these dimensions into a single composite index, rather than analyzing them separately, offering a potentially more holistic and interpretable representation of defensive pressure in dynamic match situations.

A key finding of the study is that higher DPI values were associated with more favourable defensive outcomes. Sequences characterized by higher defensive pressure were more likely to correspond to higher level defensive outcome categories. This aligns with previous research indicating that defensive pressure can disrupt attacking organization and increase the likelihood of successful defensive sequences (18, 46). Importantly, these findings suggest that this relationship can be captured through an integrated index, providing preliminary support for the DPI as a sequence level summary of defensive pressure. However, the proportional odds assumption showed evidence of deviation, indicating that the association between DPI and defensive outcomes may not be constant across outcome thresholds. This issue is addressed further in the limitations section.

In contrast, the running score had no statistically significant effect on DPI, and the effect size was negligible. This suggests that, within this sample, the defensive pressure reflected by DPI remained largely stable across different running score (losing, drawing, and winning). This could indicate that the structural components of DPI are more closely tied to the collective organization of the defense, rather than to the immediate scoreline context. While no statistically significant differences were found, a faint trend may still be observed. One possible explanation is that DPI primarily reflects local spatiotemporal interactions such as distance from the ball carrier, local density, recovery positions, and defensive line organization factors more directly influenced by positional constraints than by the broader match context. In this sense, the scoreline may influence tactical intentions, but these influences may not necessarily translate into consistent changes in defensive pressure at the sequence level. This interpretation is in line with recent studies that have highlighted the importance of spatial context in defensive performance. For example, Jamil et al. (30) showed that the effectiveness of organised pressing is influenced by spatial factors such as ball recovery location. The present findings seem consistent with this perspective, suggesting that defensive pressure is fundamentally shaped by spatial tactical elements.

At the same time, the nonsignificant effect of running score should not be interpreted as evidence that contextual factors are irrelevant. Rather, it suggests that the running score alone may be too broad a variable to capture the complexity underlying defensive behaviors. Defensive pressure is likely shaped by a wider array of situational and tactical factors that were not explicitly modelled in the present study.

This study also provides some initial insight into defensive behaviors in the CSL. Compared with patterns reported in European leagues, defensive pressure in the CSL sample appears to occur more frequently in deeper areas of the pitch, potentially suggesting a more conservative defensive approach. However, as no direct cross league comparison was conducted, this interpretation remains tentative and should be examined more systematically in future studies.

## 5 Limitations

The present study has several limitations that should be considered when interpreting the findings. First, the analyses were conducted at the level of individual defensive sequences, which were nested within matches and teams, but the models did not explicitly account for this hierarchical data structure. As a result, potential non independence among observations may have influenced standard errors and significance estimates. Future research should address this issue by using mixed effects or other hierarchical modelling approaches to better capture variation across teams and matches.

The statistical evaluation of the DPI should also be interpreted with caution. Although the index was significantly associated with defensive outcomes, the explanatory power of the model was modest, indicating that defensive performance is likely influenced by additional contextual and tactical factors not included in the present analysis. Moreover, the proportional odds assumption showed evidence of deviation, suggesting that the relationship between defensive pressure and outcomes may differ across outcome thresholds. This highlights the need for more flexible modelling approaches in future studies.

Several data related limitations should also be acknowledged. The sample was restricted to 30 matches from a single league, which may limit the generalisability of the findings to other competitions or playing contexts. In addition, the analysis focused only on organised attacking sequences with a minimum duration and multiple passes, meaning that transitional or short duration defensive situations were not captured. Finally, given the observational design of the study, the findings should be interpreted as associative rather than causal.

## 6 Future research

Future research should extend this framework by incorporating a broader range of contextual variables. Factors such as coaching strategy, tactical instructions, match period, fatigue, opponent quality, and relative team strength may all influence the application of defensive pressure.

In addition, underlying tactical structures may play an important role. Previous studies have shown that formations and positional organization can influence defensive behaviors and pressing dynamics (21). As these factors were not explicitly modelled in the present study, future research should incorporate formation and positional data to better understand their contribution to defensive pressure.

Methodologically, the use of mixed effects models, longitudinal designs, and simulation based approaches using tracking data may provide further insight into how defensive pressure evolves across different match contexts.

## 7 Conclusion

Overall, the findings suggest that defensive pressure, as captured by the Defensive Pressure Index, can be understood as a multidimensional construct emerging from collective spatial organization rather than isolated individual actions. The DPI was associated with defensive outcomes, providing preliminary evidence that it may serve as a useful sequence level summary of defensive pressure.

By contrast, DPI showed limited variation across running score, suggesting that running score alone may not adequately capture the contextual complexity underlying defensive behavior. Despite its limitations, the present study provides an operational and replicable framework for quantifying defensive pressure and offers a foundation for future research into the contextual and tactical determinants of defensive organization in professional football.

## Data Availability

The datasets presented in this article are not readily available because due to confidentiality agreements with the data provider, the dataset used in this study cannot be made publicly available. However, we have made every effort to ensure transparency and reproducibility by providing detailed descriptions of the data processing procedures and analytical methods in the manuscript. Where possible, we are willing to share additional information with the editor or reviewers upon reasonable request and subject to approval from the data provider. Requests to access the datasets should be directed to jli005@ikasle.ehu.eus.
