# Supplementary material for: Development and initial validation of a defensive pressure index using tracking data in the Chinese super league
Source: Front Sports Act Living. 2026 May 26;8:1833549. doi: 10.3389/fspor.2026.1833549 (PMC13246670; doi:10.3389/fspor.2026.1833549)
Supplement: Supplementary file 1 [file Datasheet1.pdf]

title: "21/10 DPI 1" author: "Ang" date: "2025-10-21" output: html_document

本内容为个人创作，未经许可不得使用或传播。 This work is an original creation. Unauthorized use or distribution is prohibited. 本部分代码截至第560行，后续内容为扩展部分。 The code in this section extends up to line 560, the remaining part contains extensions.

```
library(readxl)1
library(dplyr)
library(psych)
library(GGally)
library(FactoMineR)
library(factoextra)
library(nnet)
library(car)
library(DescTools)
library(janitor)

library(readxl)
total1 <- read_xlsx(file.choose())
```

```
str(total1)
summary(total1)
```

```
eps <- 1e-6
total1 <- total1 %>%
  mutate(
    DPPBND_inv  = 1 / (DPPBND + eps),
    PL_inv      = 1 / (PL + eps),
    DLAODGL_inv = 1 / (DLAODGL + eps)
  )
```

```
X <- total1 %>%
  select(DPGZ, DPPBND_inv, `Defenders Num`, PL_inv, Passes, DLAODGL_inv)
Xz <- scale(X)
R <- cor(Xz)
KMO(R)
cortest.bartlett(R, n = nrow(Xz))
pca <- prcomp(Xz, center = FALSE, scale. = FALSE)
summary(pca)
pca$rotation
total1$DPI <- pca$x[, 1]
total1$DPI_z <- as.numeric(scale(total1$DPI))
fviz_eig(pca, addlabels = TRUE)
fviz_pca_var(pca, col.var = "contrib", repel = TRUE)
```

```r
eig_vals <- pca$sdev^2
prop_var <- eig_vals / sum(eig_vals)
cum_var <- cumsum(prop_var)

eig_tab <- data.frame(
  Component = paste0("PC", seq_along(eig_vals)),
  Eigenvalue = round(eig_vals, 3),
  `Proportion of Variance` = round(prop_var, 4),
  `Cumulative Proportion`  = round(cum_var, 4)
)

eig_tab   #表的Initial Eigenvalues部分
#Varimax旋转后的loading
pc_varimax <- psych::principal(r = R, nfactors = 2, rotate = "varimax", scores = FALSE)

#loadings of each variable
load_tab <- as.data.frame(unclass(pc_varimax$loadings))
colnames(load_tab) <- c("PC1_Varimax", "PC2_Varimax")
load_tab$Variable <- rownames(load_tab)
load_tab <- load_tab[, c("Variable", "PC1_Varimax", "PC2_Varimax")]

load_tab
```

```r
#作图
#碎石图（与Figure中左上角方差解释一致）
factoextra::fviz_eig(pca, addlabels = TRUE)

#变量在PC1-PC2平面上的位置
factoextra::fviz_pca_var(
  pca, col.var = "contrib", repel = TRUE
)

#个体在 PC1-PC2 平面上的分布
#按分组着色
factoextra::fviz_pca_ind(
  pca,
  geom.ind = "point",
  habillage = total1$Outcome,
  addEllipses = TRUE,
  repel = TRUE
)
```

```
if (exists("filter", inherits = FALSE)) rm(filter)

#计算PC1/PC2的分位数范围
scores_pc12 <- as.data.frame(pca$x[, 1:2])
colnames(scores_pc12) <- c("PC1","PC2")

qx <- quantile(scores_pc12$PC1, c(0.01, 0.99), na.rm = TRUE)
qy <- quantile(scores_pc12$PC2, c(0.01, 0.99), na.rm = TRUE)
pad_x <- 0.10 * (qx[2] - qx[1])
pad_y <- 0.10 * (qy[2] - qy[1])
x_limits <- c(qx[1] - pad_x, qx[2] + pad_x)
y_limits <- c(qy[1] - pad_y, qy[2] + pad_y)

#有效样本索引（PC1/PC2有限值，分组非缺失）
hab <- factor(total1$Outcome)
idx <- is.finite(scores_pc12$PC1) &
       is.finite(scores_pc12$PC2) &
       !is.na(hab)

#个体图：缩放但不删数据，且长度一致
p_ind <- factoextra::fviz_pca_ind(
  pca,
  geom.ind    = "point",
  habillage   = hab,
  addEllipses = TRUE,
  alpha.ind   = 0.6,
  pointshape  = 21,
  pointsize   = 2,
  repel       = FALSE
) +
  coord_cartesian(xlim = x_limits, ylim = y_limits) +
  coord_fixed(ratio = 1)

print(p_ind)
```

```
unique(total1$Outcome)
```

```
total1$Outcome <- factor(total1$Outcome, levels = c("F", "GP", "CDS"))
total1$Outcome_num <- as.numeric(total1$Outcome)
model1 <- lm(Outcome_num ~ DPGZ + DPPBND_inv + `Defenders Num` + PL_inv + Passes + DLAODGL_inv, data = total1)
weights <- abs(coef(model1)[-1])
weights <- weights / sum(weights)
total1$PressureIndex <- as.matrix(Xz) %*% weights

summary(model1)
coef(model1)
weights
```

```
total1$Outcome <- factor(total1$Outcome, levels = c("F", "GP", "CDS"))
total1$Outcome_num <- as.numeric(total1$Outcome)
model1 <- lm(Outcome_num ~ DPGZ + DPPBND + `Defenders Num` + PL + Passes + DLAODGL, data = total1)
weights <- abs(coef(model1)[-1])
weights <- weights / sum(weights)
total1$PressureIndex <- as.matrix(Xz) %*% weights

summary(model1)
coef(model1)
weights
```

```r
library(ggplot2)

total1$Outcome <- factor(total1$Outcome, levels = c("F", "GP", "CDS"))

p_box <- ggplot(total1, aes(x = Outcome, y = PressureIndex, fill = Outcome)) +
  geom_boxplot(alpha = 0.8, width = 0.8, color = "black", size = 0.2) +

  scale_fill_manual(values = c(
    "F"   = "#D9333B",
    "GP"  = "#096DD9",
    "CDS" = "#FFEB6B"
  )) +

  labs(
    x = "Outcome",
    y = "Pressure Index"
  ) +

  theme_minimal(base_size = 12) +
  theme(
    plot.title = element_text(hjust = 0.5, face = "bold", size = 12),
    axis.title.x = element_text(hjust = 0.5, size = 12),
    axis.title.y = element_text(hjust = 0.5, size = 12),
    text = element_text(family = "Times New Roman")
  )

p_box


jpeg("box_outcome_300dpi.jpg",
     width = 6, height = 4, units = "in",
     res = 300)

print(p_box)

dev.off()
```

```r
ggplot(total1, aes(x = PressureIndex, fill = Outcome)) +
  geom_density(alpha = 0.5) +
  labs(
    title = "Distribution of Pressure Index across Defensive Outcomes",
    x = "Pressure Index",
    y = "Density"
  ) +
  theme_minimal(base_size = 14)
```

```r
library(MASS)
total1$Outcome <- ordered(total1$Outcome, levels = c("F", "GP", "CDS"))

eps <- 1e-3
total1 <- total1 %>%
  mutate(
    DPPBND_inv = 1 / (DPPBND + eps),
    PL_inv     = 1 / (PL + eps),
    DLAODGL_inv= 1 / (DLAODGL + eps)
  ) %>%
  mutate(across(ends_with("_inv"), ~ pmin(., quantile(., 0.99, na.rm=TRUE)))) %>%
  mutate(across(ends_with("_inv"), scale))

fit_polr <- polr(Outcome ~ DPGZ + DPPBND_inv + `Defenders Num` + PL_inv + Passes + DLAODGL_inv,
                 data = total1, method = "logistic", Hess = TRUE)

summary(fit_polr)
```

```r
library(ggplot2)
class(fit_polr)
```

```r
coef_summary <- coef(summary(fit_polr))
coef_df <- as.data.frame(coef_summary)
coef_df$Variable <- rownames(coef_df)

colnames(coef_df) <- c("Estimate", "StdError", "tValue", "Variable")

coef_df <- subset(coef_df, !(Variable %in% c("F|GP", "GP|CDS")))

library(ggplot2)

ggplot(coef_df, aes(x = reorder(Variable, Estimate), y = Estimate, fill = Estimate > 0)) +
  geom_col(width = 0.6, show.legend = FALSE) +
  geom_errorbar(aes(ymin = Estimate - StdError, ymax = Estimate + StdError), width = 0.2) +
  geom_text(aes(label = round(Estimate, 3)),
            hjust = ifelse(coef_df$Estimate > 0, -0.3, 1.3), size = 4) +
  coord_flip() +
  scale_fill_manual(values = c("#e74c3c", "#3498db")) +
  labs(title = "Coefficients of Ordered Logistic Regression (Pressure Success Model)",
       x = "Variables",
       y = "Coefficient (log-odds)") +
  theme_minimal(base_size = 14)
```

```r
library(MASS)

fit_polr_nopass <- polr(as.factor(Outcome) ~ DPGZ + DPPBND_inv + `Defenders Num` +
                          PL_inv + DLAODGL_inv,
                        data = total1, method = "logistic", Hess = TRUE)
AIC(fit_polr_nopass)

fit_polr_nopl <- polr(as.factor(Outcome) ~ DPGZ + DPPBND_inv + `Defenders Num` +
                        Passes + DLAODGL_inv,
                      data = total1, method = "logistic", Hess = TRUE)
AIC(fit_polr_nopl)
```

```r


total1$Outcome <- factor(total1$Outcome,
                         levels = c("F", "GP", "CDS"),
                         ordered = TRUE)

fit_polr_inv <- polr(as.factor(Outcome) ~ DPGZ + DPPBND_inv + `Defenders Num` +
                       PL_inv + Passes + DLAODGL_inv,
                     data = total1, method = "logistic", Hess = TRUE)
AIC(fit_polr_inv)
```

```r
total1$Outcome <- factor(total1$Outcome,
                         levels = c("F", "GP", "CDS"),
                         ordered = TRUE)

fit_polr_raw <- polr(as.factor(Outcome) ~ DPGZ + DPPBND + `Defenders Num` +
                       PL + Passes + DLAODGL,
                     data = total1, method = "logistic", Hess = TRUE)
AIC(fit_polr_raw)
```

```r
AIC(fit_polr_inv, fit_polr_raw)
```

```r
summary(fit_polr_raw)
```

```
 #dpi outcome
needed_pkgs <- c("MASS", "brant", "pscl")
to_install <- needed_pkgs[!sapply(needed_pkgs, requireNamespace, quietly = TRUE)]

if (length(to_install) > 0) {
  install.packages(to_install)
}

library(MASS)
library(brant)
library(pscl)

#F < GP < CDS
total1$Outcome <- factor(
  total1$Outcome,
  levels = c("F", "GP", "CDS"),
  ordered = TRUE
)

table(total1$Outcome)

#构造 DPI
#DPI = -Z(DPGZ) - Z(DPPBND) - Z(DNUM) + Z(PL) - Z(PN) + Z(DLAODGL)
z_dpgz      <- as.numeric(scale(total1$DPGZ))
z_dppbnd    <- as.numeric(scale(total1$DPPBND))
z_dnum      <- as.numeric(scale(total1$`Defenders Num`))
z_pl        <- as.numeric(scale(total1$PL))
z_passes    <- as.numeric(scale(total1$Passes))
z_dlaodgl   <- as.numeric(scale(total1$DLAODGL))

total1$DPI <- -z_dpgz - z_dppbnd - z_dnum + z_pl - z_passes + z_dlaodgl

summary(total1$DPI)
sd(total1$DPI, na.rm = TRUE)

#有序logistic回归 Outcome~DPI
fit_dpi_polr <- polr(
  Outcome ~ DPI,
  data = total1,
  method = "logistic",
  Hess = TRUE
)

summary(fit_dpi_polr)

#提取系数、标准误、p值、OR、95%CI
#polr 默认不给p值，这里用Wald z检验近似
coef_table <- coef(summary(fit_dpi_polr))

#只取DPI这一行
beta <- coef_table["DPI", "Value"]
se   <- coef_table["DPI", "Std. Error"]
z    <- coef_table["DPI", "t value"]
p    <- 2 * (1 - pnorm(abs(z)))
#OR
OR <- exp(beta)
#95% Wald CI for beta and OR
lower_beta <- beta - 1.96 * se
upper_beta <- beta + 1.96 * se

lower_OR <- exp(lower_beta)
upper_OR <- exp(upper_beta)
#结果
cat("Beta =", round(beta, 4), "\n")
cat("SE =", round(se, 4), "\n")
cat("z =", round(z, 4), "\n")
```

```
cat("z =", round(z, 4), "\n")
cat("p =", signif(p, 4), "\n")
cat("OR =", round(OR, 4), "\n")
cat("95% CI for OR = [", round(lower_OR, 4), ", ", round(upper_OR, 4), "]\n", sep = "")
#整理成表
result_main <- data.frame(
  Predictor = "DPI",
  Beta = beta,
  SE = se,
  z_value = z,
  p_value = p,
  OR = OR,
  CI_low = lower_OR,
  CI_high = upper_OR
)

result_main

#McFadden pseudo-R2
pR2_values <- pscl::pR2(fit_dpi_polr)
pR2_values

#单独取McFadden
mcfadden_r2 <- pR2_values["McFadden"]
cat("McFadden pseudo-R2 =", round(as.numeric(mcfadden_r2), 4), "\n")
#比例优势假设（proportional odds assumption）
#用 brant 检验 若 overall p > 0.05，通常认为没有明显违反
brant_result <- brant::brant(fit_dpi_polr)
brant_result

#预测概率 后面作图解释可能要用
newdat <- data.frame(
  DPI = seq(min(total1$DPI, na.rm = TRUE),
            max(total1$DPI, na.rm = TRUE),
            length.out = 100)
)

pred_prob <- predict(fit_dpi_polr, newdata = newdat, type = "probs")
pred_df <- cbind(newdat, pred_prob)

head(pred_df)
```

```
names(total1)
```

```
#version2
needed_pkgs <- c("MASS", "brant", "pscl")
to_install <- needed_pkgs[!sapply(needed_pkgs, requireNamespace, quietly = TRUE)]
if (length(to_install) > 0) install.packages(to_install)
library(MASS)
library(brant)
library(pscl)

#删失
dat_dpi <- total1[complete.cases(total1[, c("DPGZ", "DPPBND", "Defenders Num", "PL", "Passes", "DLAODGL", "Outcome")]), ]

#F < GP < CDS
dat_dpi$Outcome <- factor(
  dat_dpi$Outcome,
  levels = c("F", "GP", "CDS"),
  ordered = TRUE
)

table(dat_dpi$Outcome)

#DPI
```

```
#DPI
z_dpgz    <- as.numeric(scale(dat_dpi$DPGZ))
z_dppbnd  <- as.numeric(scale(dat_dpi$DPPBND))
z_dnum    <- as.numeric(scale(dat_dpi$`Defenders Num`))
z_pl      <- as.numeric(scale(dat_dpi$PL))
z_passes  <- as.numeric(scale(dat_dpi$Passes))
z_dlaodgl <- as.numeric(scale(dat_dpi$DLAODGL))
dat_dpi$DPI <- -z_dpgz - z_dppbnd - z_dnum + z_pl - z_passes + z_dlaodgl
summary(dat_dpi$DPI)
sd(dat_dpi$DPI, na.rm = TRUE)

#有序logistic回归
fit_dpi_polr <- polr(
  Outcome ~ DPI,
  data = dat_dpi,
  method = "logistic",
  Hess = TRUE
)
summary(fit_dpi_polr)

#提取系数与OR
coef_table <- coef(summary(fit_dpi_polr))

beta <- coef_table["DPI", "Value"]
se   <- coef_table["DPI", "Std. Error"]
z    <- coef_table["DPI", "t value"]
p    <- 2 * (1 - pnorm(abs(z)))

OR <- exp(beta)
lower_beta <- beta - 1.96 * se
upper_beta <- beta + 1.96 * se
lower_OR <- exp(lower_beta)
upper_OR <- exp(upper_beta)

result_main <- data.frame(
  Predictor = "DPI",
  Beta = beta,
  SE = se,
  z_value = z,
  p_value = p,
  OR = OR,
  CI_low = lower_OR,
  CI_high = upper_OR
)

print(result_main)

#McFadden pseudo-R2
pR2_values <- pscl::pR2(fit_dpi_polr)
print(pR2_values)

mcfadden_r2 <- pR2_values["McFadden"]
cat("McFadden pseudo-R2 =", round(as.numeric(mcfadden_r2), 4), "\n")

#Brant 检验
brant_result <- brant::brant(fit_dpi_polr)
print(brant_result)

#预测概率
newdat <- data.frame(
  DPI = seq(min(dat_dpi$DPI, na.rm = TRUE),
            max(dat_dpi$DPI, na.rm = TRUE),
            length.out = 100)
)

pred_prob <- predict(fit_dpi_polr, newdata = newdat, type = "probs")
```

```
pred_df <- cbind(newdat, pred_prob)

head(pred_df)
```

```
 #anova_model <- aov(DPI ~ Running score)

#TukeyHSD和空格列名 导致内部模型矩阵解析失败，有bug
#重命名变量
names(total1)[names(total1) == "Running score"] <- "RunningScore"

table(total1$RunningScore, useNA = "ifany")
total1$RunningScore <- factor(
  total1$RunningScore,
  levels = c("Losing", "Drawing", "Winning")
)

#ANOVA
anova_model <- aov(DPI ~ RunningScore, data = total1)
summary(anova_model)

#Tukey
tukey_result <- TukeyHSD(anova_model)
tukey_result

#η2
anova_tab <- summary(anova_model)[[1]]

ss_between <- anova_tab[1, "Sum Sq"]
ss_within  <- anova_tab[2, "Sum Sq"]

eta2 <- ss_between / (ss_between + ss_within)
eta2
```

```r
#total1$Outcome <- factor(total1$Outcome, levels = c("F","GP","CDS"), ordered = TRUE)
library(MASS)
fit_polr_raw <- polr(Outcome ~ DPGZ + DPPBND + `Defenders Num` + PL + Passes + DLAODGL,
                     data = total1, method = "logistic", Hess = TRUE)

summary(fit_polr_raw)
null_fit <- update(fit_polr_raw, . ~ 1)
#A
anova(null_fit, fit_polr_raw, test = "Chisq")
#B
library(lmtest)
lrtest(null_fit, fit_polr_raw)

library(brant)
brant(fit_polr_raw)

vars <- c("DPGZ","DPPBND","`Defenders Num`","PL","Passes","DLAODGL")

lr_per_var <- lapply(vars, function(v){
  fm_red <- as.formula(paste("Outcome ~", paste(setdiff(vars, v), collapse = " + ")))
  fit_red <- update(fit_polr_raw, fm_red)
  out <- lrtest(fit_red, fit_polr_raw)
  data.frame(var = v,
             Chisq = unname(out$Chisq[2]),
             df    = unname(out$Df[2]),
             p     = unname(out$`Pr(>Chisq)`[2]))
})
lr_table <- do.call(rbind, lr_per_var)
lr_table[order(lr_table$p), ]

coefs <- coef(fit_polr_raw)
ses   <- sqrt(diag(vcov(fit_polr_raw)))[names(coefs)]
OR_tab <- data.frame(
  term   = names(coefs),
  OR     = exp(coefs),
  CI_low = exp(coefs - 1.96*ses),
  CI_hi  = exp(coefs + 1.96*ses),
  row.names = NULL
)
OR_tab

LL_full <- as.numeric(logLik(fit_polr_raw))
LL_null <- as.numeric(logLik(null_fit))
McFadden_R2 <- 1 - (LL_full / LL_null)
McFadden_R2

# install.packages("VGAM")
# library(VGAM)
# fit_parallel <- vglm(Outcome ~ DPGZ + DPPBND + `Defenders Num` + PL + Passes + DLAODGL,
#                       family = cumulative(link = "logit", parallel = TRUE), data = total1)
# fit_nonpar  <- vglm(Outcome ~ DPGZ + DPPBND + `Defenders Num` + PL + Passes + DLAODGL,
#                      family = cumulative(link = "logit", parallel = FALSE), data = total1)
# lrtest(fit_parallel, fit_nonpar)
#对违背假设的变量放宽partial PO
```

```
nums <- c("DPGZ","DPPBND","Defenders Num","PL","Passes","DLAODGL","Own_score","Scor_difference")
pairs(total1[ , nums], pch = 19, cex = 0.4)

cors <- sapply(c("Own_score","Scor_difference"), function(y){
  sapply(c("DPGZ","DPPBND","Defenders Num","PL","Passes","DLAODGL"), function(x){
    c(pearson = suppressWarnings(cor(total1[[y]], total1[[x]], use="complete.obs", method="pearson")),
      spearman = suppressWarnings(cor(total1[[y]], total1[[x]], use="complete.obs", method="spearman")))
  })
})
round(cors, 3)
```

```
library(MASS)
library(lmtest)

rhs <- ~ DPGZ + DPPBND + `Defenders Num` + PL + Passes + DLAODGL

bad_rows <- which(!is.finite(total1$Own_score) | total1$Own_score < 0)
cat("Own_score 非法行数：", length(bad_rows), "\n")
if (length(bad_rows) > 0) {
  cat("示例问题行（前10行）:\n")
  print(head(total1[bad_rows, c("Own_score","Scor_difference")], 10))
}

dat_goal <- subset(total1, is.finite(Own_score) & Own_score >= 0)

vars <- c("Own_score","DPGZ","DPPBND","Defenders Num","PL","Passes","DLAODGL")
dat_goal <- dat_goal[stats::complete.cases(dat_goal[ , vars]), ]

cat("用于进球模型的样本量：", nrow(dat_goal), "\n")

fml_pois <- as.formula(paste("Own_score", deparse(rhs)))
m_pois <- glm(fml_pois, data = dat_goal, family = poisson())
cat("\n=== Poisson 回归 ===\n"); print(summary(m_pois))

dispersion <- sum(residuals(m_pois, type = "pearson")^2) / m_pois$df.residual
cat("\nDispersion =", round(dispersion, 3), "\n")

if (dispersion > 1.5) {
  m_qp <- glm(fml_pois, data = dat_goal, family = quasipoisson())
  cat("\n=== quasi-Poisson ===\n"); print(summary(m_qp))

  m_nb <- glm.nb(fml_pois, data = dat_goal)
  cat("\n=== Negative Binomial ===\n"); print(summary(m_nb))

  cat("\nAIC:  Poisson =", AIC(m_pois),
      ";  NegBin =", AIC(m_nb), "\n")
}

co <- coef(m_pois)
se <- sqrt(diag(vcov(m_pois)))
OR_tab_goals <- data.frame(
  term   = names(co),
  OR     = exp(co),
  CI_low = exp(co - 1.96*se),
  CI_hi  = exp(co + 1.96*se),
  row.names = NULL
)
cat("\n=== 进球数模型：比值比(OR)与95%CI（Poisson）===\n"); print(OR_tab_goals)
R_tab_goals
```

```
total1$Match_Status <- with(total1,
                            ifelse(Scor_difference > 0, "Winning",
                                   ifelse(Scor_difference == 0, "Drawing", "Losing")))

total1$Match_Status <- factor(total1$Match_Status,
                              levels = c("Losing", "Drawing", "Winning"))

table(total1$Match_Status)
head(total1[, c("Own_score", "Scor_difference", "Match_Status")])
```

```
need <- c("ggplot2","car","effectsize","FSA","dplyr")
to_install <- setdiff(need, rownames(installed.packages()))
if (length(to_install)>0) install.packages(to_install, quiet = TRUE)
invisible(lapply(need, library, character.only = TRUE))

total1$Match_Status <- with(total1,
                            ifelse(Scor_difference > 0, "Winning",
                                   ifelse(Scor_difference == 0, "Drawing", "Losing")))
total1$Match_Status <- factor(total1$Match_Status,
                              levels = c("Losing","Drawing","Winning"))

cat("各状态样本量：\n"); print(table(total1$Match_Status))

pressure_col <- NULL
cand <- c("PressureIndex","Pressure","pressure","pressure_index")
for (nm in cand) if (nm %in% names(total1)) { pressure_col <- nm; break }

if (is.null(pressure_col)) {
  message("未发现现成的压力指标列；临时构造 PressureIndex_z（标准化后求和）用于分析。")
  vars_press <- c("DPGZ","DPPBND","Defenders Num","PL","Passes","DLAODGL")
  miss <- setdiff(vars_press, names(total1))
  if (length(miss)>0) stop(paste("缺少变量：", paste(miss, collapse=", ")))

  Z <- scale(total1[ , vars_press])
  total1$PressureIndex_z <- rowSums(Z, na.rm = FALSE)
  pressure_col <- "PressureIndex_z"
}

cat("本次分析使用的压力指标列：", pressure_col, "\n")

dat <- total1 %>%
  dplyr::select(all_of(pressure_col), Match_Status) %>%
  dplyr::filter(is.finite(.data[[pressure_col]]), !is.na(Match_Status))
names(dat)[1] <- "PressureIndex"

cat("可用于分析的样本量：", nrow(dat), "\n")

desc <- dat %>%
  group_by(Match_Status) %>%
  summarise(n = n(),
            mean = mean(PressureIndex),
            sd = sd(PressureIndex),
            se = sd/sqrt(n),
            ci95_low = mean - qt(0.975, df=n-1)*se,
            ci95_hi  = mean + qt(0.975, df=n-1)*se,
            .groups="drop")
cat("\n描述统计（均值±95%CI）：\n"); print(desc)

p1 <- ggplot(dat, aes(x = Match_Status, y = PressureIndex, fill = Match_Status)) +
  geom_boxplot(alpha = 0.75, outlier.alpha = 0.5) +
  labs(x = "Match Status", y = "Pressure Index",
       title = "Defensive Pressure by Match Status") +
  theme_minimal() + theme(legend.position = "none")
print(p1)
```

```r
aov_fit <- aov(PressureIndex ~ Match_Status, data = dat)
cat("\nANOVA 结果：\n"); print(summary(aov_fit))

resid_ok <- TRUE
if (nrow(dat) <= 5000) {
  sh <- shapiro.test(residuals(aov_fit))
  cat("\n残差Shapiro-Wilk正态性检验：W=", round(sh$statistic,3),
      ", p=", signif(sh$p.value,3), "\n", sep="")
  resid_ok <- (sh$p.value > 0.05)
} else {
  cat("\n样本较大，正态性采用图形诊断（Q-Q）更合适。\n")
}

dat$PressureIndex <- as.numeric(dat$PressureIndex)
dat$Match_Status  <- droplevels(factor(dat$Match_Status))

lev <- car::leveneTest(y = dat$PressureIndex,
                       group = dat$Match_Status,
                       center = median)
print(lev)
var_equal <- (lev[1, "Pr(>F)"] > 0.05)

eta <- effectsize::eta_squared(aov_fit, partial = FALSE)
cat("\n效应量（Eta-squared）：\n"); print(eta)

if (summary(aov_fit)[[1]][["Pr(>F)"]][1] < 0.05) {
  post <- TukeyHSD(aov_fit)
  cat("\nTukey 事后比较：\n"); print(post)
}

if (!resid_ok || !var_equal) {
  cat("\nANOVA假设不充分，补充非参数检验：Kruskal–Wallis\n")
  kw <- kruskal.test(PressureIndex ~ Match_Status, data = dat)
  print(kw)
  if (kw$p.value < 0.05) {
    cat("\nDunn 事后比较（Bonferroni）：\n")
    print(FSA::dunnTest(PressureIndex ~ Match_Status, data = dat, method = "bonferroni"))
  }
}
```

```r
p2 <- ggplot(desc, aes(x = Match_Status, y = mean, fill = Match_Status)) +

  geom_col(width = 0.8, color = NA, alpha = 0.8) +

  geom_errorbar(
    aes(ymin = ci95_low, ymax = ci95_hi),
    width = 0.15,
    size = 0.3
  ) +

  scale_fill_manual(values = c(
    "Losing"  = "#D9333B",
    "Drawing" = "#096DD9",
    "Winning" = "#FFEB6B"
  )) +

  labs(
    x = "Running Score",
    y = "Mean Pressure Index",
    fill = "Running Score"
  ) +

  theme_minimal(base_size = 12) +
  theme(
    plot.title = element_text(hjust = 0.5, face = "bold", size = 12),
    axis.title.x = element_text(hjust = 0.5, size = 12),
    axis.title.y = element_text(hjust = 0.5, size = 12),
    text = element_text(family = "Times New Roman"),
    legend.position = "right"
  )

p2



jpeg("p2_300dpi.jpg",
     width = 6, height = 4, units = "in",
     res = 300)

print(p2)

dev.off()
```

```r
need <- c("dplyr","car","effectsize","emmeans","parameters","performance",
          "sandwich","lmtest","GGally","ggplot2","relaimpo","mgcv")
to_install <- setdiff(need, rownames(installed.packages()))
if(length(to_install)>0) install.packages(to_install, quiet=TRUE)
invisible(lapply(need, library, character.only=TRUE))
```

```r
install.packages("relaimpo", repos = "https://cloud.r-project.org")
```

```r
need <- c("dplyr","car","effectsize","emmeans","parameters","performance",
          "sandwich","lmtest","GGally","ggplot2","relaimpo","mgcv")
to_install <- setdiff(need, rownames(installed.packages()))
if(length(to_install)>0) install.packages(to_install, repos="https://cloud.r-project.org", quiet=TRUE)
invisible(lapply(need, library, character.only=TRUE))

stopifnot(exists("total1"))

if(!("Match_Status" %in% names(total1))){
  if(!("Scor_difference" %in% names(total1))) stop("缺少 Scor_difference，无法构造 Match_Status。")
  total1$Match_Status <- with(total1, ifelse(Scor_difference > 0, "Winning",
                                      ifelse(Scor_difference == 0, "Drawing", "Losing")))
```

```
}
total1$Match_Status <- factor(total1$Match_Status, levels=c("Losing","Drawing","Winning"))

pressure_col <- NULL
for(nm in c("PressureIndex","pressure","Pressure","pressure_index")){
  if(nm %in% names(total1)) { pressure_col <- nm; break }
}
if(is.null(pressure_col)){
  message("未检测到现成 PressureIndex，临时以六变量的z分数和构造 PressureIndex_z。")
  vars_press <- c("DPGZ","DPPBND","Defenders Num","PL","Passes","DLAODGL")
  miss <- setdiff(vars_press, names(total1))
  if(length(miss)>0) stop(paste("缺少变量：", paste(miss, collapse=", ")))
  Z <- scale(total1[, vars_press])
  total1$PressureIndex_z <- rowSums(Z)
  pressure_col <- "PressureIndex_z"
}

dat <- total1 |>
  dplyr::select(all_of(pressure_col), Match_Status, DPGZ, DPPBND, `Defenders Num`, PL, Passes, DLAODGL) |>
  dplyr::filter(is.finite(.data[[pressure_col]]))
names(dat)[1] <- "PressureIndex"

scale_cols <- c("DPGZ","DPPBND","Defenders Num","PL","Passes","DLAODGL")
dat[scale_cols] <- lapply(dat[scale_cols], scale)

dat$Match_Status <- droplevels(factor(dat$Match_Status, levels=c("Losing","Drawing","Winning")))
cat("样本量：", nrow(dat), "；各状态分布：\n"); print(table(dat$Match_Status))

m_base <- lm(PressureIndex ~ DPGZ + DPPBND + `Defenders Num` + PL + Passes + DLAODGL + Match_Status, data=dat)
m_int  <- lm(PressureIndex ~ (DPGZ + DPPBND + `Defenders Num` + PL + Passes + DLAODGL) * Match_Status, data=dat)

cat("\n[模型比较] 是否需要交互：\n"); print(anova(m_base, m_int))
cat("\n[Type-III 联合检验] 各变量 × 比分状态 交互：\n"); print(car::Anova(m_int, type=3))

cat("\n[稳健标准误 HC3] 交互模型系数：\n")
print(lmtest::coeftest(m_int, vcov = sandwich::vcovHC(m_int, type="HC3")))

for(ref in c("Losing","Drawing","Winning")){
  tmp <- dat; tmp$Match_Status <- stats::relevel(tmp$Match_Status, ref=ref)
  fit <- lm(PressureIndex ~ (DPGZ + DPPBND + `Defenders Num` + PL + Passes + DLAODGL) * Match_Status, data=tmp)
  cat("\n=== 以", ref, "为参照的组内斜率（标准化β±95%CI）===\n")
  out <- parameters::model_parameters(fit, effects="fixed", ci=0.95)
  print(dplyr::filter(out, grepl("^(DPGZ|DPPBND|Defenders Num|PL|Passes|DLAODGL)($|:)", Parameter)))
}

scale_cols <- c("DPGZ","DPPBND","Defenders Num","PL","Passes","DLAODGL")
to_z_numeric <- function(x) as.numeric(scale(x))

dat_fix <- dat
dat_fix[scale_cols] <- lapply(dat_fix[scale_cols], to_z_numeric)
dat_fix$Match_Status <- droplevels(factor(dat_fix$Match_Status,
                                          levels = c("Losing","Drawing","Winning")))

m_int_fix <- lm(
  PressureIndex ~ (DPGZ + DPPBND + `Defenders Num` + PL + Passes + DLAODGL) * Match_Status,
  data = dat_fix
)

emm <- emmeans::emmeans(m_int_fix, ~ Match_Status)
print(emm)

nd <- expand.grid(
  PL = seq(min(dat_fix$PL, na.rm = TRUE), max(dat_fix$PL, na.rm = TRUE), length.out = 80),
  Match_Status = levels(dat_fix$Match_Status)
)
```

```
nd$Match_Status <- factor(nd$Match_Status, levels = levels(dat_fix$Match_Status))
nd$DPGZ <- 0; nd$DPPBND <- 0; nd$`Defenders Num` <- 0; nd$Passes <- 0; nd$DLAODGL <- 0
nd <- nd[, c("PL","Match_Status","DPGZ","DPPBND","Defenders Num","Passes","DLAODGL")]

nd$fit <- predict(m_int_fix, newdata = nd)

p_slp <- ggplot(nd, aes(x = PL, y = fit, color = Match_Status)) +
  geom_line(size = 1.1) +
  scale_color_manual(
    name = "Running Score",
    values = c(
      "Losing"  = "#D9333B",
      "Drawing" = "#096DD9",
      "Winning" = "#FFEB6B"
    )
  ) +
  labs(
    x = "Pass length (PL)",
    y = "Predicted Defensive Pressure",
  ) +
  theme_minimal(base_size = 12) +
  theme(
    plot.title = element_text(hjust = 0.5, face = "bold", size = 12),
    axis.title.x = element_text(size = 12),
    axis.title.y = element_text(size = 12),
    text = element_text(family = "Times New Roman"),
    legend.position = "right"
  )

p_slp



jpeg("p_slp_300dpi.jpg",
     width = 6, height = 4, units = "in",
     res = 300)

print(p_slp)

dev.off()
```

```
get_importance <- function(d){
  fit <- lm(PressureIndex ~ DPGZ + DPPBND + `Defenders Num` + PL + Passes + DLAODGL, data=d)
  relaimpo::calc.relimp(fit, type=c("lmg"))$lmg |> sort(decreasing=TRUE)
}
imp_L <- get_importance(subset(dat, Match_Status=="Losing"))
imp_D <- get_importance(subset(dat, Match_Status=="Drawing"))
imp_W <- get_importance(subset(dat, Match_Status=="Winning"))
cat("\n[相对重要性 lmg] Losing：\n");  print(round(imp_L,3))
cat("\n[相对重要性 lmg] Drawing：\n"); print(round(imp_D,3))
cat("\n[相对重要性 lmg] Winning：\n"); print(round(imp_W,3))

dat_gam <- dat
dat_gam$Defenders_Num <- dat_gam$`Defenders Num`

dat_gam$Match_Status <- droplevels(factor(dat_gam$Match_Status,
                                          levels = c("Losing","Drawing","Winning")))

gam_fit <- mgcv::gam(
  PressureIndex ~ Match_Status +
    s(PL, by = Match_Status, k = 5) +
    s(DLAODGL, by = Match_Status, k = 5) +
    s(DPGZ, k = 5) + s(DPPBND, k = 5) + s(Defenders_Num, k = 5) + s(Passes, k = 5),
  data   = dat_gam,
  method = "REML"
)
```

```
cat("\n[GAM 摘要]：\n"); print(summary(gam_fit))
cat("\n[GAM 方差解释与AIC]：\n"); print(performance::model_performance(gam_fit))

n_panels <- length(gam_fit$smooth)
smooth_terms <- vapply(gam_fit$smooth, function(x) x$term, FUN.VALUE = "")

labels_clean <- c(
  "Losing - PL",
  "Drawing - PL",
  "Winning - PL",
  "Losing - DLAODGL",
  "Drawing - DLAODGL",
  "Winning - DLAODGL",
  "DPGZ",
  "DPPBND",
  "Defenders_Num",
  "Passes"
)

jpeg("gam_fitx_600dpi.jpg",
     width = 9, height = 12, units = "in",
     res = 600)

par(mfrow = c(4, 3),
    family = "Times")
for(i in seq_len(n_panels)){

  xlab_i <- smooth_terms[i]
  ylab_i <- labels_clean[i]

  if(smooth_terms[i] == "Defenders_Num"){
    xlab_i <- "DNUM"
    ylab_i <- "DNUM"
  }

  if(smooth_terms[i] == "Passes"){
    xlab_i <- "PN"
    ylab_i <- "PN"
  }

  plot(gam_fit,
       select = i,
       scheme = 1,
       shade = TRUE,
       seWithMean = TRUE,
       xlab = xlab_i,
       ylab = ylab_i,
       cex = 1.0,
       cex.lab = 1.0,
       cex.axis = 1.0,
       font.lab = 1,
       font.axis = 1)
}

dev.off()

cat("\n—— 分析完成：\n",
    "1) anova(m_base, m_int) 判断是否需要交互；\n",
    "2) Type-III 显著的交互项 => 哪些变量随比分改变作用；\n",
    "3) 组内斜率表 => 在 Losing/Drawing/Winning 下的标准化β；\n",
    "4) 相对重要性(lmg) => 每种比分下变量解释度排序；\n",
    "5) GAMM => 检查并可视化非线性与形状差异。\n")
```

```
rmarkdown::render("R1.Rmd")
```
