# Supplementary material for: Development and initial validation of a defensive pressure index using tracking data in the Chinese super league
Source: Front Sports Act Living. 2026 May 26;8:1833549. doi: 10.3389/fspor.2026.1833549 (PMC13246670; doi:10.3389/fspor.2026.1833549)
Supplement: Supplementary file 2 [file Table1.docx]

Appendix: PCA results for DPI variables.

| Panel A. Component loadings and communalities | | | | | |
| --- | --- | --- | --- | --- | --- |
| Variable | MSA | PC1 | PC2 | | Communality(PC1-PC2) |
| DPGZ | 0.51 | 0.674 | -0.078 | | 0.46 |
| DPPBND | 0.53 | -0.052 | 0.153 | | 0.03 |
| DNUM | 0.50 | -0.056 | -0.696 | | 0.49 |
| PL | 0.49 | -0.267 | -0.648 | | 0.49 |
| PN | 0.55 | 0.459 | -0.050 | | 0.21 |
| DLAODGL | 0.52 | 0.508 | -0.254 | | 0.33 |
| Panel B. Model diagnostics | | | | | |
| Statistic | | | | Value | |
| KMO (overall) | | | | 0.51 | |
| Bartlett’s test χ²(15) | | | | 344.37*** | |
| Variance explained (PC1) | | | | 22.95% | |
| Variance explained (PC2) | | | | 20.13% | |
| Cumulative variance | | | | 43.08% | |

*Note:* MSA = measure of sampling adequacy. Only components with eigenvalues > 1 are interpreted. *** p < 0.001.
